# AI Increases the Pressure to Overhaul the Scientific Peer Review Process. Comment on “Artificial Intelligence Can Generate Fraudulent but Authentic-Looking Scientific Medical Articles: Pandora’s Box Has Been Opened”

**DOI:** 10.2196/50591

**Published:** 2023-08-31

**Authors:** Nicholas Liu, Amy Brown

**Affiliations:** 1 John A Burns School of Medicine University of Hawai'i at Mānoa Honolulu, HI United States; 2 Department of Quantitative Health Sciences John A Burns School of Medicine University of Hawai'i at Mānoa Honolulu, HI United States

**Keywords:** artificial intelligence, AI, publications, ethics, neurosurgery, ChatGPT, Chat Generative Pre-trained Transformer, language models, fraudulent medical articles

Májovský and colleagues’ [1] concern regarding OpenAI’s ChatGPT is valid. Seven months ago, the release of ChatGPT was quickly tempered by warnings of perpetuating biases and spreading misinformation. Artificial intelligence (AI) tools threaten to amplify preexisting issues in academic publishing, particularly the scientific peer review process. This outdated system is overwhelmed by the volume of new journals and papers produced by a growing global academic community [2], a problem that AI is fit to accentuate. Here are three areas in need of modification:

The scientific peer review process

- The body of qualified reviewers is drowning in a rising sea of writers that includes top researchers, undergraduate students, and academics at all levels in between [2].
- Peer review lacks formal standards or guidelines, as well as training, particularly in statistics [3], creating a restricted and top-heavy pool of qualified reviewers [2].
- Reviewers are declining to perform reviews more often since the notion of reviewing as a professional obligation fails to sufficiently recognize or reward the burden it imposes [2].
- Peer review fraud, which involves conflicts of interest, influence, and false identities, has evolved out of a need for reviews.

Publication pressure

- The dearth of reviewers is compounded by the proliferation of plagiarized, fraudulent, and otherwise low-quality work [4].
- Pressure to “publish or perish” has led to high-profile cases of academic fraud and likewise feeds “paper mills” that churn out questionable research for academics who are desperate to progress in their careers [4].
- The proliferation of “for-profit” journals subverts respectful publishing through financialization that exploits and alienates scientists [2].

AI integration

- Májovský et al [1] displayed the effectiveness of ChatGPT as an open access ghostwriter, capable of fabricating a complete and convincing article in just one hour.
- In one study, only 63% of ChatGPT-generated abstracts were caught by reviewers as fakes [5]. In response to such findings, Science is updating its license and editorial policies to prohibit AI-generated text, figures, or graphics [5].
- Such staunch resistance is misguided; AI may not be an author per se, but its utility in all stages of research, from generating topics and compiling information to writing text, cannot be ignored. If an AI-generated, human-reviewed paper communicates quality research, why should it be disallowed? Moreover, how would we tell?
- Although AI-generated text detection software can help [1], detection bypass tools are similarly available online.

AI makes the need for high-quality peer reviews greater and more pressing than ever before. The cornerstone of scientific integrity is on the path to obsoletion without a viable successor. As academic pursuits become increasingly inseparable from industry, conceptualizing peer review as a duty to science will no longer suffice. Respecting and empowering the peer review system will involve considering reviewers as expert consultants, performing reviews as productive work, and creating system-wide guidelines that integrate (rather than resist) AI technologies. This problem, emerging from an imperative for success, needs a peer review system and publication process that has more teeth than trust, a commodity that served us well in the past, but whose restoration bears reinvention.

## Abbreviations

AI: artificial intelligence
